# Pyruvate metabolism is involved in adaptability and cariogenicity of *Streptococcus mutans*

**DOI:** 10.1080/20002297.2026.2612843

**Published:** 2026-01-11

**Authors:** Shuxing Yu, Yaqi Liu, Dingwei Ye, Xinyue Wang, Yumeng Wu, Jing Zou, Qizhao Ma

**Affiliations:** a State Key Laboratory of Oral Diseases & National Center for Stomatology & National Clinical Research Center for Oral Diseases, West China Hospital of Stomatology, Sichuan University, Chengdu, Sichuan, People's Republic of China; b Department of Pediatric Dentistry, West China Hospital of Stomatology, Sichuan University, Chengdu, Sichuan, People's Republic of China

**Keywords:** Dental caries, *Streptococcus mutans*, pyruvate, biofilms, oxidative stress, metabolic regulation

## Abstract

Dental caries is a biofilm-mediated disease that arises from polymicrobial dysbiosis in dental plaque. Among these microorganisms, *Streptococcus mutans* plays a prominent role because of its strong capacity to metabolize fermentable carbohydrates into organic acids that drive enamel demineralization. Central to this process is pyruvate, a key metabolic intermediate that connects glycolysis, energy production, biosynthesis, and stress adaptation. Pyruvate metabolism in *S. mutans* directs carbon flow into various pathways that contribute to its cariogenic potential, including acidogenesis, biofilm formation, and oxidative stress tolerance. This review explores the multifaceted roles of pyruvate in *S. mutans*, emphasizing its involvement in the production of lactate, acetate, formate, and branched-chain amino acids. We also discuss the regulatory mechanisms that control pyruvate metabolism, such as the Pta-Ack pathway, LrgAB-mediated pyruvate transport, and transcriptional regulation by CcpA/CodY. Furthermore, we highlight promising strategies for caries prevention, including the targeting of pyruvate metabolism using natural compounds and metabolic inhibitors. Future research should focus on elucidating the regulatory networks governing pyruvate metabolism, the metabolic byproducts, and the impact of disrupting pyruvate-based metabolic crosstalk in polymicrobial biofilms. Understanding how pyruvate functions as a carrier or precursor metabolite in central carbon metabolism of *S. mutans* and its regulation of survival and metabolic processes will have significant implications for caries prevention.

## Introduction

Dental caries remains one of the most common chronic diseases worldwide, posing a significant public health challenge despite advances in dental science and preventive measures [1]. The aetiology of dental caries involves complex interactions among host factors, dietary components, and cariogenic bacteria, with *Streptococcus mutans* identified as a primary microbial agent in the pathogenesis of this disease [2]. The ability of *S. mutans* to metabolise fermentable carbohydrates into acid is a cornerstone of its cariogenic potential, which leads to tooth enamel demineralisation and subsequent cavity formation [3]. Importantly, this pathogenic capacity is best appreciated within the broader ecological framework of oral streptococci [4]. Health-associated early colonisers such as *Streptococcus sanguinis*, *Streptococcus gordonii*, *Streptococcus mitis*, and *Streptococcus oralis* share the core glycolytic machinery with *S. mutans* but generally contribute to community stability through less persistently acidifying physiology and stronger competitive or pH-buffering traits, including H₂O₂-associated antagonism and alkali-generating systems that help restrain plaque acidification [5,6]. In contrast, *S. mutans* is particularly well-adapted to sustain high glycolytic throughput and remain metabolically active under low-pH conditions, enabling it to dominate in dysbiotic, carbohydrate-rich biofilms [7].

The central metabolic pathway in *S. mutans* involves the conversion of sugars to pyruvate via glycolysis, followed by further fermentation to lactate [8,9]. While pyruvate is a universal metabolic intermediate, it is particularly important for *S. mutans* and other cariogenic oral streptococci because their ecological success in plaque depends on sustained glycolytic ATP production and acidogenic fermentation under carbohydrate-rich conditions [10,11]. In this context, pyruvate functions as a decisive branch-point metabolite that not only fuels lactate-dominant acid production but also supports redox homoeostasis and stress adaptation needed for persistence in mature biofilms [12,13]. Moreover, the ability of *S. mutans* to redistribute and re-assimilate extracellular pyruvate provides an additional layer of metabolic flexibility that can influence oxidative stress tolerance and competitive interactions within polymicrobial communities [12,14]. Thus, in cariogenic streptococci, pyruvate links carbohydrate availability to acidification, survival under acid stress, and biofilm-associated virulence, making it a particularly relevant node for understanding and potentially targeting cariogenic physiology.

The metabolism of pyruvate in *S. mutans* is highly adaptive, allowing the bacterium to thrive in the fluctuating environment of the oral cavity. Under anaerobic conditions, which are prevalent in the dense biofilms of dental plaque, pyruvate is converted into lactate, the primary acid implicated in the initiation and progression of dental caries [13]. However, pyruvate also feeds into the tricarboxylic acid cycle (TCA) and other aerobic pathways when more oxygen is available, demonstrating the metabolic flexibility of *S. mutans* [12,14]. This flexibility, together with its exceptional tolerance to environmental acidification, helps explain why *S. mutans* is more likely than many other oral streptococci to persist and remain acidogenic in mature cariogenic biofilms.

This review explores the multifaceted roles of pyruvate in the metabolic strategies of *S. mutans*, focusing on its production through glycolysis, its involvement in energy production, and its contribution to the synthesis of critical virulence factors such as organic acids and biofilm components. Additionally, we delve into the regulatory mechanisms that control pyruvate metabolism and discuss how these pathways interact with environmental factors to influence the cariogenic potential of *S. mutans*. By dissecting the pathways and regulatory networks surrounding pyruvate metabolism in *S. mutans*, this review aims to highlight innovative strategies that could mitigate or even prevent the metabolic processes responsible for caries development, offering new avenues for the management and prevention of this widespread dental disease.

### Carbohydrate metabolism and pyruvate production

The metabolic processes of different organisms vary widely, yet pyruvate serves as a common precursor metabolite essential for the formation of all biomass [15]. *S. mutans*, known for its ability to thrive in the challenging environment of dental plaque and contribute to the caries process, exhibits broad carbohydrate catabolic capacity that supports its persistence in fluctuating oral nutrient conditions. Phenotypic and genomic evidence indicates that *S. mutans* can utilise a wide range of common dietary and host-associated carbohydrates, including multiple mono-, di-, and oligosaccharides, thereby sustaining robust glycolytic flux and continuous pyruvate supply in plaque biofilms [16]. While many health-associated oral streptococci also share core sugar catabolic routes, *S. mutans* is distinguished by its efficient sucrose-driven EPS production, strong acidogenicity, and superior aciduricity, features that collectively promote persistent biofilm maturation and localised enamel demineralisation [17].

*S. mutans* incorporates sugars into its metabolism primarily through two systems: the phosphotransferase (PTS) system, which is phosphoenolpyruvate (PEP)-dependent, and non-PTS transporters that rely on binding proteins or permeases for sugar uptake [18]. This dual transport capacity enables *S. mutans* to utilise a broad spectrum of carbohydrates, including glucose, fructose, sucrose, lactose, and raffinose-family oligosaccharides, as well as various plant-derived glycosides [17,19]. Once inside the cytoplasm, these carbohydrates are funneled into central carbon metabolism, with key intermediates such as fructose-6-phosphate entering pathways that support glycolysis and cell wall synthesis [16]. The PTS system in particular can transport multiple disaccharides and glycosides (e.g. cellobiose, salicin, arbutin, and aesculin), many of which contain a glucose moiety linked to another sugar or substituent, thereby expanding metabolic flexibility under fluctuating nutrient conditions [20]. Beyond serving as a major route for carbohydrate entry, the PTS system is tightly integrated with global regulatory networks that coordinate carbon flow and virulence [17,21–24]. By coupling sugar transport to PEP consumption, PTS activity directly shapes the cellular PEP/pyruvate balance and thereby influences metabolic decisions at the pyruvate branch-point. This transport–metabolism coupling interfaces with carbon catabolite regulatory circuits, including both CcpA-associated and CcpA-independent mechanisms, to prioritise preferred carbohydrates and fine-tune expression of catabolic and fermentative genes [21,23]. These systems converge with the Cid/Lrg module and the LrgAB pyruvate transporter, which contributes to pyruvate uptake and stationery-phase physiology, further connecting carbohydrate availability to pyruvate homoeostasis and persistence in biofilms [17,24].

**Figure 1.**
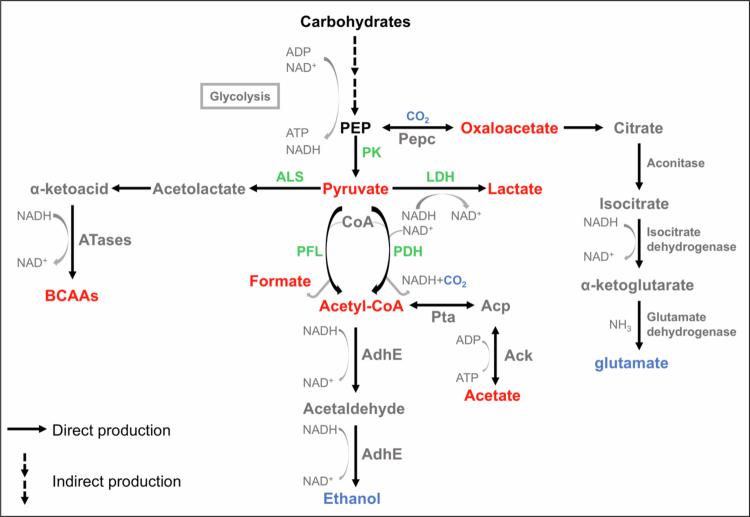
Schematic representation of the pyruvate sources and metabolic pathway in *Streptococcus mutans*. Pyruvate, derived from glucose and other sugars via glycolysis, serves as a central metabolic intermediate. Pyruvate is converted into several metabolites, including lactate, acetyl-CoA, formate, and BCAAs through ATases. Acetyl-CoA participates in the tricarboxylic acid (TCA) cycle or is converted to Acp by Pta, and subsequently to acetate by Ack. ALS also contributes to BCAA synthesis. Additionally, acetaldehyde is produced through the action of AdhE. PEP: phosphoenolpyruvate; Acetyl-CoA: acetyl coenzyme A; BCAAs: branched-chain amino acids; Acp: acetyl phosphate; Pepc: PEP carboxylase; PK: pyruvate kinase; LDH: lactate dehydrogenase; PFL: pyruvate formate lyase; PDH: pyruvate dehydrogenase; ALS: acetolactate synthase; Pta: phosphotransacetylase; Ack: acetate kinase; ATases: aminotransferases. AdhE: alcohol-acetaldehyde dehydrogenase. Colour scheme: red highlights key pyruvate-derived intermediates; green denotes key enzymes required for the formation of these functionally important intermediates; and blue represents by-products generated during pyruvate metabolism.

During glycolysis, fructose-6-phosphate (fructose-6P) is converted through a series of enzyme-catalysed reactions to phosphoenolpyruvate (PEP). The 6-phosphofructokinase PfkA phosphorylates fructose-6P to fructose-1,6-bisphosphate (fructose-1,6P2), a key regulatory step in glycolysis. Fructose-1,6-bisphosphate aldolase (FbaA) then cleaves this intermediate into two three-carbon molecules: dihydroxyacetone phosphate (DHAP) and glyceraldehyde-3-phosphate (G3P). Triosephosphate isomerase converts DHAP into G3P, ensuring both molecules enter the same downstream pathway. Glyceraldehyde-3-phosphate dehydrogenase (GapC) catalyses the oxidation of G3P to 1,3-bisphosphoglycerate, coupled with the reduction of NAD⁺ to NADH. Subsequent steps, including those catalysed by phosphoglycerate kinase and phosphoglycerate mutase, ultimately lead to the formation of 2-phosphoglycerate. Enolase (Eno) then dehydrates 2-phosphoglycerate to form PEP. This series of reactions results in ATP and NADH production, which are critical for cellular energy metabolism in *S. mutans* [25–27]. The final and rate-limiting step of this pathway is catalysed by pyruvate kinase (PK), which transfers a phosphate from PEP to adenosine diphosphate (ADP), forming pyruvate and ATP (Figure 1). This conversion not only provides energy in the form of ATP but also generates pyruvate, a key metabolic junction crucial for further biosynthetic and energy-producing reactions within *S. mutans* [28].

### Major routes of pyruvate metabolism

Pyruvate, a pivotal intermediate in glycolysis, serves as the precursor for numerous cellular components and products via diverse metabolic pathways. In the Emden-Meyerhof-Parnas (EMP) pathway, pyruvate is converted into acetyl coenzyme A (acetyl-CoA). The metabolism of acetyl-CoA in *S. mutans* exemplifies a complex and interconnected network involving glucose metabolism, fatty acid metabolism, amino acid metabolism, among other processes. These metabolic pathways collectively sustain the normal physiological functions and proliferation of *S. mutans*, enabling its adaptation and survival within the oral environment.

Furthermore, pyruvate undergoes a metabolic journey involving decarboxylation through various enzymatic reactions, including those catalysed by lactate dehydrogenase (LDH), pyruvate formate lyase (PFL), pyruvate dehydrogenase (PDH), and acetolactate synthase (ALS). These enzymatic processes convert pyruvate into a diverse array of metabolites, such as formate, lactate, oxaloacetate, and branched-chain amino acids (Figure 1). These pathways are essential not only for energy production but also for amino acid biosynthesis and numerous other vital biosynthetic routes.

#### Pyruvate and formate formation

Under anaerobic and carbohydrate-limited conditions, *S. mutans* can route pyruvate through pyruvate formate lyase (PFL) to generate formate and acetyl-CoA [29,30]. This branch supports fermentative metabolism and helps balance redox demands when oxygen is scarce. Formate has been detected at relatively high levels in resting dental plaque and may serve as a community metabolite in polymicrobial biofilms, particularly under nutrient-restricted conditions where PFL activity is favoured [31,32].

#### Pyruvate and acetate formation

When oxygen is available, the pyruvate dehydrogenase (PDH) complex catalysers the conversion of pyruvate to acetyl-CoA with concomitant NADH production [25,29]. Acetyl-CoA can be further directed to acetate via the Pta-Ack pathway, generating acetyl phosphate (AcP), a metabolite with both energetic and regulatory significance [33]. This branch contributes to metabolic flexibility and links carbon flux to stress adaptation and signalling.

#### Pyruvate and lactate formation

Under conditions of sufficient carbohydrate availability, lactate dehydrogenase (LDH) undergoes allosteric activation by fructose-1,6P2, enhancing its catalytic efficiency in converting pyruvate to lactate. This LDH-driven pyruvate-to-lactate flux is a hallmark of anaerobic metabolism in *S. mutans*, supporting glycolytic ATP production by regenerating NAD⁺ when oxygen is limited [34–36]. The lactate produced during this process accumulates intracellularly and diffuses into the extracellular milieu, thereby lowering environmental pH. Such acidification promotes the cariogenic potential of *S. mutans* by reinforcing its survival and metabolic activity under acidic conditions and facilitating enamel demineralisation.

#### Pyruvate and oxaloacetate formation

Under glycolytic conditions, the end products of glycolysis—PEP and pyruvate—feed into TCA-related reactions primarily through conversion of pyruvate to acetyl-CoA [6]. Although *S. mutans* possesses an incomplete TCA cycle, pyruvate- and PEP-dependent anaplerotic reactions remain important for maintaining intracellular pools of key carbon intermediates required for biosynthetic balance. Oxaloacetate can be generated from PEP via PEP carboxylase (PC) and PEP carboxykinase (PCK), or from pyruvate via pyruvate carboxylase [10,33]. These routes supply substrates for amino acid and other anabolic pathways and thereby connect central carbon metabolism with growth and stress resilience [37,38].

#### Pyruvate and branched-chain amino acid formation

Proteomic analysis of *S. mutans* under acidic growth conditions revealed elevated levels of proteins associated with branched-chain amino acid (BCAA) biosynthesis. Under aerobic conditions, ALS catalyses the conversion of pyruvate to acetolactate [39]. This intermediate is transformed into *α*-ketoacid, which is then converted to BCAAs such as isoleucine, leucine, and valine through a series of reactions mediated by aminotransferases (ATases). These amino acids are integral to bacterial protein structure and function. The metabolism of acetolactate may proceed to form acetoin, which is catalysed by acetolactate decarboxylase, ultimately leading back to acetyl-CoA and acetaldehyde, thus closing a critical metabolic loop [40–43].

### Transport of pyruvate to the extracellular environment

Pyruvate, a versatile metabolite, can be channelled into various metabolic pathways or stored as an energy reserve when in surplus. As the nexus of bacterial energy metabolism and biosynthesis, pyruvate may enter the TCA cycle for aerobic respiration or be converted into organic acids under hypoxic conditions. *S. mutans*, possessing only a truncated TCA cycle and devoid of cytochrome components, relies primarily on the EMP pathway for ATP generation. Within this pathway, fructose-6P undergoes a series of enzymatic transformations to yield pyruvate [29,36,44]. The dynamics of pyruvate metabolism in *S. mutans* are closely linked to the availability of carbon sources. During conditions of carbon sufficiency, overflow metabolism facilitates rapid bacterial growth by producing energy and lactate, with the concurrent release of excess pyruvate from cells [36,45,46]. The excretion (overflow) of pyruvate is a common feature of many bacteria when cultured under excess carbon conditions, contributing to the metabolic balance between carbon uptake and consumption [47–50]. In bacteria, the efflux of pyruvate may depend on the bacterial efflux pump system. These efflux pumps are usually composed of membrane protein complexes that are able to transport different kinds of substances from the inside of the cell to the outside [51,52]. Although the mechanism of pyruvate expulsion in *S. mutans* is not well understood, these proteins may utilise mechanisms such as concentration gradients, electrochemical potentials, and other dynamic processes to facilitate the transport of pyruvate or its derivatives from the intracellular to the extracellular environment. However, the precise structures and functions of these specific transport proteins remain to be elucidated through further investigation.

Conversely, under carbon-limited conditions, the uptake of extracellular pyruvate may support prolonged ATP production via the pyruvate transporter LrgAB and could serve as a secondary carbon source that may help mitigate cell lysis during the stationary phase [53]. Similar LrgAB-dependent pyruvate uptake has been reported in *B. subtilis* and *S. aureus*, indicating that this adaptive strategy may be conserved among Gram-positive bacteria [54,55]. This process contributes to the stabilisation of cellular processes, prevents the detrimental effects of carbon starvation, and promotes a more balanced and efficient utilisation of available resources. In essence, the strategic utilisation of extracellular pyruvate under carbon-limited conditions represents a significant adaptive strategy that bolsters cellular endurance and functional robustness.

### Regulatory mechanisms of metabolic fluxes at the pyruvate branch-point

**Figure 2.**
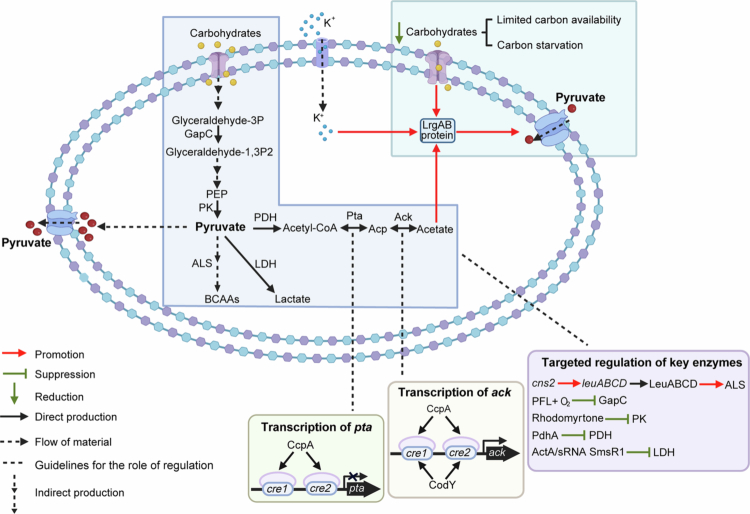
Regulation and metabolic flux of pyruvate in *Streptococcus mutans*. During growth, pyruvate is excreted as a spillover metabolite, while during nutrient starvation, it is reabsorbed. Pyruvate uptake via the LrgAB transporter is critical for *S. mutans* during hypoglycaemia stress. Inactivation of the Pta-Ack pathway completely abolishes LrgAB activity and the organism’s ability to absorb environmental pyruvate, while the inactivation of the PFL pathway also impairs the uptake of pyruvate during the stationary phase. Under transcriptional regulation, CcpA binds to two potential *cre* sites in the *ack* promoter, while CodY binds to the CodY box, overlapping the *cre2* site of *ack*. CodY binding is dependent on the availability of specific amino acids, particularly BCAAs, which enhance CodY binding. When glycolytic intermediates accumulate, CcpA negatively regulates *pta* expression by binding directly to two possible *cre* sites in the *pta* promoter region. Additionally, acetate and potassium (K^+^)are suggested to serve as environmental or metabolic signals that enable *S. mutans* cells to sense and respond to increased external pyruvate levels before these increases are regulated by their own internal feedback mechanisms.

To fine-tune carbon flux at the pyruvate branch-point, *S. mutans* employs a suite of regulatory mechanisms that ensure metabolic flexibility in response to nutrient availability and environmental stress. These mechanisms govern the direction and utilisation of pyruvate toward energy generation, biosynthesis, and adaptive responses. Among them, the Pta-Ack pathway and the LrgAB-mediated pyruvate transport system are two critical nodes that integrate intracellular metabolic signals with environmental cues (Figure 2).

#### Pta-Ack pathway

The optimisation of acetate metabolism in *S. mutans* enhances adaptation to fluctuations in carbohydrate and amino acid availability, as well as environmental pH changes, through the regulation of the Pta-Ack pathway. This pathway indicates a complex metabolic linkage between pyruvate uptake, acetate production, stress tolerance, biofilm development, and broader metabolic adaptations that warrants further investigation [56].

The interaction of catabolite response protein A (CcpA) and CodY with the *ackA* and *pta* promoters significantly influences the Pta-Ack pathway, affecting the overall carbon flux and acetate production in *S. mutans*. CcpA involved in carbon catabolite repression (CCR), binds to the catabolite-responsive element (*cre*) to dynamically modulate gene expression [57]. CodY is a global transcriptional regulator that is conserved in many low G + C Gram-positive bacteria, including *S. mutans*. It facilitates rapid adaptation to changes in nutrient availability and contributes to physiological homoeostasis by sensing intracellular levels of branched-chain amino acids (BCAAs) [58–60]. Research indicates that both CcpA and CodY are direct regulators of *ackA* and *pta* gene transcription in *S. mutans* [56]. CcpA and CodY exert a synergistic effect on the transcriptional regulation of *ackA*, enhancing its expression to adapt to the metabolic needs [49]. Deactivation of either CcpA or CodY in *S. mutans* leads to a decrease in *ackA* promoter activity, demonstrating their essential roles in activating this gene's transcription. Conversely, the inactivation of CcpA uniquely results in increased activity of the *pta* promoter, suggesting a compensatory regulatory mechanism that adjusts the production of acetyl phosphate to maintain metabolic balance [56]. Importantly, CodY specifically binds to the *ackA* promoter region in the presence of BCAAs, indicating a nutrient-responsive regulatory mechanism that integrates amino acid signals into metabolic gene regulation [49,59,61,62]. This regulation is crucial for the ability of *S. mutans* to adapt to varying nutrient levels and environmental conditions, ensuring efficient energy production and survival in the oral cavity.

Additionally, the Pta-Ack pathway in *S. mutans* plays a crucial role in maintaining stable levels of acetyl phosphate (AcP), serving both as a signalling molecule and a phosphate donor within two-component systems (TCSs), thus bridging central metabolism with environmental sensing and signal transduction. This pathway significantly impacts stress tolerance, biofilm development, and the metabolism of guanosine pentaphosphate and tetraphosphate (ppGpp), which are critical elements in bacterial stress responses [61].

The synthesis of intracellular AcP through the Pta-Ack pathway enhances the ability of *S. mutans* to tolerate oxidative stress and contributes to biofilm formation, which is pivotal for its survival in the oral cavity. The pathway involves the *pta* gene, which is part of the four-gene *relQ* operon [63]. During oxidative stress, the inactivation of the Pta-Ack pathway affects both *relQ* and *relP*, leading to decreased accumulation of (p)ppGpp, which is vital for the response of *S. mutans* to ROS produced by its own endogenous metabolic processes or competing microorganisms [33].

#### Transport of pyruvate

The transport of pyruvate at the onset of stationary phase is mediated by the holin/antiholin-like protein LrgAB. The LrgAB operon is activated under low glucose conditions to absorb expelled pyruvate and is suppressed when glucose levels are high [64]. This regulation allows bacteria to utilise overflow pyruvate, enhancing survival and metabolic activity as cells enter the stationary phase, a process coordinated by the LytST two-component regulatory system (TCS) [53].

Furthermore, the PFL enzyme, which is inactivated in the presence of oxygen, plays a crucial role in the metabolic adaptation of *S. mutans* under nutritional stress by affecting the reuptake of pyruvate during the stationary phase [65]. Environmental factors such as acetate and potassium (K^+^) are significant in modulating the LrgAB-mediated pyruvate uptake, potentially acting as metabolic signals that enable *S. mutans* to sense and respond to fluctuations in external pyruvate levels [66]. The disruption of the Pta-Ack pathway, which converts acetyl-CoA to acetate, results in a lack of induction of the LrgAB system in the stationary phase, thus affecting pyruvate sensing and uptake [65].

### Impact of pyruvate and its metabolites on bacterial biological functions

Pyruvate metabolism is a pivotal hub in central carbon metabolism. In the oral microbiota, pyruvate-derived pathways are tightly linked to cariogenic ecology, particularly in *S. mutans*, whose success in plaque depends on sustained carbohydrate catabolism and downstream virulence expression [1,2]. As a central carbon node, pyruvate supports bacterial proliferation and energy generation [10,11,67]. Under low-glucose conditions, it can serve as an alternative carbon source that sustains acetyl-CoA production and redox balance via the coordinated actions of PDH and PFL [29,30]. Consistent with this role, exogenous pyruvate has been shown to extend the exponential growth phase of *S. mutans* and increase biomass, suggesting that pyruvate availability may help sustain metabolic activity under carbon limitation [65]. Beyond growth fitness, pyruvate-centred fluxes also shape major virulence-associated traits of *S. mutans*, including biofilm formation, acidogenicity, aciduricity, and oxidative stress adaptability.

#### Pyruvate involved in biofilm formation of S. mutans

Biofilm formation is central to the cariogenic lifestyle of *S. mutans*, enabling persistence on tooth surfaces and promoting localised acidification within structured plaque communities [68,69]. Because biofilm development is highly energy- and carbon-intensive, pyruvate-centred metabolism is well positioned to influence this process by coordinating glycolytic output with the supply of precursors and reducing equivalents required for extracellular polysaccharide (EPS) synthesis and community expansion. In particular, pyruvate kinase (PK) governs the terminal step of glycolysis and thereby shapes the availability of pyruvate and downstream acetyl-CoA pools that can support biosynthetic demands during biofilm growth [70]. Consistent with this framework, higher PK activity has been associated with increased colony abundance and greater biomass in dental plaque biofilms, suggesting that robust glycolytic-to-pyruvate flux contributes to the colonisation fitness and structural stability of *S. mutans* biofilms [71].

In addition to flux control, emerging evidence indicates that post-translational regulation may connect pyruvate-derived acetyl donors to the enzymatic machinery of matrix production. The acetyltransferase ActG can acetylate glucosyltransferases (Gtfs) in *S. mutans* using acetyl-CoA, limiting the formation of water-insoluble EPS and reducing biofilm biomass [72,73]. These findings indicate that pyruvate-derived metabolites not only fuel energy production but also modulate biofilm-related enzyme activity, linking central metabolism with virulence regulation.

#### Pyruvate involved in acidogenicity and aciduricity of S. mutans

The primary virulence factors of *S. mutans—*acid production (acidogenicity) and the ability to survive at low pH (aciduricity)*—*are closely tied to its metabolism, particularly the catabolism of pyruvate. This metabolic intermediate is central to the regulation of pathways that not only govern the production of organic acids but also enhance the acid resistance in *S. mutans*, thereby influencing its cariogenic potential.

The utilisation of sugar alcohols, such as sorbitol, as sugar substitutes in food products has been a significant strategy to prevent caries. Sorbitol is particularly valued in the confectionery industry due to its low fermentability and favourable physicochemical properties [74]. Metabolic studies indicate that sorbitol's metabolism leads to an increased oxidation of PFL and an elevated NADH/NAD^+^ ratio. This biochemical shift inhibits glyceraldehyde-phosphate dehydrogenase (GapC), thereby reducing the phosphorylation and oxidation of glyceraldehyde-3P. As a result, acid production by *S. mutans* is curtailed, particularly in thinner dental plaque biofilms, effectively reducing caries incidence [27].

A critical enzyme, pyruvate dehydrogenase A (PdhA)—the E1 *α*-subunit of the PDH complex—plays a role in the heterocatalytic conversion of pyruvate to acetyl-CoA [75]. Research has demonstrated that PdhA is involved in heterofermentation, which influences the aciduricity of *S. mutans*. The activity of PdhA directly impacts the aciduricity of *S. mutans*, suggesting that targeting PdhA could offer new strategies to reduce acid tolerance and cariogenic properties [8].

The metabolism of amino acids also presents an alternative pathway for *S. mutans* to mitigate the effects of acidic end products. The synthesis of BCAAs such as isoleucine, leucine, and valine from pyruvate acts as an overflow pathway that not only reduces the production of acidic end products but also alleviates intracellular acidification [39]. The biosynthesis of BCAAs, particularly isoleucine, produces NH3^+^, which reacts with protons to form ammonia, helping to neutralise the acidic environment within the cytoplasm and improving the pH balance and enhancing cellular survival under acidic stress [42].

During periods of stress induced by acidic conditions, *S. mutans* can redistribute or regulate its metabolites to lessen the burden of organic acid production [76]. Proteomic analysis under such conditions has revealed changes in the expression of metabolic proteins linked to central carbon metabolism, especially those involved in BCAA biosynthesis [42]. The alteration of carbon flux from acid production to BCAA synthesis may represent a significant physiological adaptation that enhances acid tolerance and enables *S. mutans* to outcompete other oral species [77]. Notably, the genes related to BCAA formation, such as *leuA*, *leuB*, *leuC*, and *leuD*, are significantly downregulated in the *csn2* gene deletion strains, enhancing their acid tolerance. The *csn2* gene, a component of the clustered regularly interspaced short palindromic repeats (CRISPRs), appears to be intricately linked to the acid adaptation processes of *S. mutans* [78].

Acetylation modification plays a subtle and crucial regulatory role in bacterial metabolism and pathogenesis [79]. The potential mechanism of lysine acetylation may also contribute to the precise regulation of the activity of proteins related to acid tolerance and acid production in *S. mutans* [80]. Currently, the acetyltransferase ActA and sRNA SmsR1 have been identified in *S. mutans*, which can directly or indirectly acetylate the lysine sites of LDH enzyme, inhibit its activity, and ultimately reduce the acidogenicity and aciduricity of *S. mutans* [81,82].

#### Pyruvate involved in oxidative stress adaptability of S. mutans

The oxidative stress response mechanism of *S. mutans* is crucial for its survival and pathogenicity. In the oral environment, bacteria frequently encounter threats from ROS generated by the host immune system, ingested food, and other microorganisms. Pyruvate plays a significant role in the oxidative stress responses of oral microbes by reacting with ROS, particularly hydrogen peroxide (H₂O₂), to produce various byproducts that buffer against exogenous oxidative stress [5,53,83,84]. In *S. mutans*, pyruvate alleviates the cell wall stress induced by antibiotics and protects the *adh* mutant [85]. Notably, the ecological relevance of this function becomes clearer when contrasted with health-associated oral streptococci. Some commensal oral streptococci utilise pyruvate oxidase to generate H₂O₂, which inhibits the growth of *S. mutans*, while *S. mutans* itself releases pyruvate to eliminate H₂O₂ and prevent its own growth. In this process, the hypothetical water-forming NADH dehydrogenase (Nox) is a key component necessary for pyruvate release and oxidative protection [5,86–88]. Furthermore, when co-cultured with bacteria that produce H₂O₂, pyruvate aids multiple strains in competing for survival [5]. These contrasting strategies suggest that *S. mutans* leverages pyruvate not only as a metabolic intermediate but also as a defensive currency that buffers peroxide-driven antagonism in polymicrobial biofilms.

In the future, dental caries prevention and treatment are anticipated to be achieved through the regulation of pyruvate metabolism. An inhibitor could be designed to selectively block the oxidation protection mechanism of *S. mutans* via pyruvate, without affecting beneficial oral streptococci, thereby reducing its pathogenicity. Pyruvate is essential for the energy metabolism and environmental stress response of *S. mutans*. Concurrently, enhancing the inhibitory effect of symbiotic oral streptococci on *S. mutans* growth through probiotics and other means also shows significant potential. Promoting the growth and metabolism of symbiotic bacteria to produce more inhibitory substances can suppress the number and activity of *S. mutans*, leading to a healthier balance in the oral microbial community, reducing the risk of caries, and offering new strategies for maintaining oral health.

### The potential of modulating pyruvate metabolism in caries prevention and treatment

**Figure 3.**
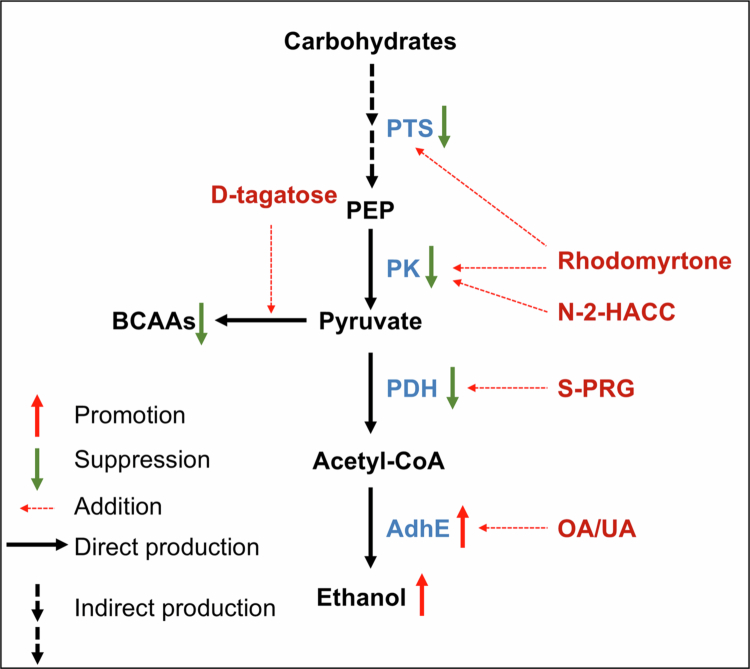
Regulation of pyruvate metabolism by natural extracts and compounds. Pyruvate metabolism is closely linked to the glycolytic pathway, with PTS facilitating sugar uptake. Pyruvate is generated from PEP and is involved in key metabolic pathways, including the synthesis of acetyl-CoA and ethanol. Various compounds, such as D-tagatose, rhodomyrtone, N-2-HACC, and S-PRG, impact the activity of pyruvate metabolism by inhibiting key enzymes, including PK, PDH, and acetate production. In contrast, OA and UA promote ethanol production via increased activity of AdhE. PTS: phosphotransferase; OA/UA: oleanolic acid/ursolic acid; S-PRG: surface pre-reacted glass-ionomer; N-2-HACC: N-2-Hydroxypropyl trimethyl ammonium chloride chitosan. Colour scheme: blue denotes key enzymes involved in pyruvate metabolism and red labels natural products or compounds that target and regulate these enzymes.

Given the central role of pyruvate metabolism in the growth and virulence of *S. mutans*, modulation of pyruvate-centred pathways may represent a promising direction for ecological caries control. However, it should be noted that many currently reported anticaries agents were not originally designed to specifically target pyruvate metabolism; rather, their effects on pyruvate availability, glycolytic output, or pyruvate-branch enzymes may reflect downstream or pleiotropic consequences of broader antibacterial or stress-related mechanisms (Figure 3).

Several natural compounds have been reported to reduce acid production or impair metabolic fitness in *S. mutans*, with accompanying changes in pyruvate-related processes. For example, oleanolic acid (OA) and ursolic acid (UA) exhibit anticaries activity and may influence central carbon metabolism and redox homoeostasis, which could secondarily affect pyruvate utilisation and downstream fermentation [89]. Rhodomyrtone has also been shown to suppress acidogenic outcomes, with evidence implicating glycolytic components such as PTS and PK; nevertheless, the extent to which these represent direct molecular targets versus broader metabolic consequences remains to be fully defined [90].

In addition, clinically relevant ionic or biomaterial-based strategies may intersect with pyruvate metabolism. Sodium fluoride (NaF) and surface pre-reacted glass-ionomer (S-PRG) eluates have been reported to alter expression of genes associated with central metabolic pathways, including pyruvate-related nodes, which may contribute to reduced growth and acid production [91,92]. Likewise, D-tagatose and N-2-Hydroxypropyl trimethyl ammonium chloride chitosan (N-2-HACC) appear to inhibit *S. mutans* fitness with measurable effects on pyruvate-associated metabolic outputs, though the precise mechanistic hierarchy linking these observations requires further clarification [93,94].

Collectively, these findings support the concept that pyruvate-centred metabolism constitutes a tractable vulnerability in *S. mutans*, while also underscoring the need for future studies using targeted genetic, biochemical, and metabolomic approaches to establish specificity and causal mechanisms for pyruvate-directed interventions.

## Conclusion

Pyruvate metabolism in *S. mutans* serves as a central hub connecting glycolysis, energy production, biosynthesis, and stress adaptation, underpinning its cariogenic virulence. As a metabolic intersection, pyruvate directs carbon flux toward pathways critical for acidogenesis, biofilm formation, and oxidative stress tolerance. The production of lactate, acetate, and formate via pyruvate-derived pathways directly contributes to enamel demineralisation, while its role in branched-chain amino acid (BCAA) synthesis and acetyl phosphate (AcP) signalling enhances aciduricity and biofilm resilience. Regulatory mechanisms, including the Pta-Ack pathway, LrgAB-mediated pyruvate transport, and transcriptional control by CcpA/CodY, highlight the adaptability of *S. mutans* to fluctuating oral environments. Targeting pyruvate metabolism has already shown promise in caries prevention, with natural compounds (e.g. oleanolic acid, rhodomyrtone) and ionic agents (e.g. fluoride) disrupting acidogenicity, biofilm formation, and oxidative stress resilience.

However, gaps remain in our understanding of pyruvate’s role as a signalling molecule and its integration with global regulatory networks. Future studies should integrate multi-omics (transcriptomics, metabolomics, proteomics) to map pyruvate’s regulatory networks under diverse environmental stresses, including nutrient limitation and interspecies competition. Assess how pyruvate cross-feeding within polymicrobial biofilms influences caries progression and whether disrupting metabolic crosstalk can mitigate dysbiosis. Investigating the functions and fates of metabolic byproducts such as formate, acetate, and branched-chain amino acids in the pyruvate metabolic process would provide a more comprehensive understanding of the mechanisms involved. Additionally, high-throughput screening of natural compounds and synthetic analogues could yield potent, selective inhibitors of pyruvate-metabolising enzymes with minimal impact on the commensal microbiota. Combinatorial strategies—such as coupling metabolic inhibitors with probiotics that enhance H₂O₂-producing oral streptococci—may synergistically suppress *S. mutans* dominance.

In conclusion, pyruvate metabolism represents a flexible and sophisticated node in the pathogenesis of *S. mutans*. By unravelling and exploiting the metabolic Achilles’ heel of *S. mutans*, it will unlock innovative, mechanism-based strategies to disrupt cariogenicity while preserving oral microbial homoeostasis.
